# Effects of *N*-acetylcysteine on the erythrocyte and liver cholinesterase, nitric oxide and malondialdehyde levels in acute organophosphate toxicity

**DOI:** 10.1186/cc9786

**Published:** 2011-03-11

**Authors:** A Bayır, H Kara, Ö Köylü, R Kocabaş, A Ak

**Affiliations:** 1 Selçuk University, Selçuklu Faculty of Medicine, Konya, Turkey; 2 Konya State Hospital, Konya, Turkey; 3 Meram Educating and Training Hospital, Konya, Turkey; 4 Selçuk University, Meram Faculty of Medicine, Konya, Turkey

## Introduction

The aim of this study was to investigate the effects of *N*-acetylcysteine (NAC) on the levels of erythrocyte and liver cholinesterase (CE), nitric oxide (NO) and malondialdehyde (MDA) in acute organophosphate poisoning (AOP) and to compare with pralidoxime (PAM)-atropine treatment.

## Methods

Twenty rabbits were divided into sham (*n *= 8), PAM-atropine (*n *= 6), and NAC groups (*n *= 6). The basal blood samples were taken from each test subject to measure plasma and erythrocyte CE, NO, and MDA values before toxicity. All of the groups were given 50 mg/kg DDVP orogastrically. The rabbits in the sham group did not receive treatment. The test subjects in the PAM-atropine and NAC groups were given 0.05 mg/kg atropine with repeated doses when required and 30 mg/kg i.v. bolus, then 15 mg/kg PAM i.v. every 4 hours. In addition to PAM and atropine, the NAC group received 30 mg/kg NAC i.v. every 6 hours. Blood samples were taken from the rabbits in all groups in the first, 12th and 24th hours to measure plasma CE, NO and MDA. Laparatomy was performed on all subjects in the 24th hour and liver tissue samples were obtained to evaluate CE, NO and MDA values in the tissues.

## Results

The erythrocyte CE levels of the NAC group were considerably higher than the sham and PAM-atropine groups in the 12th hour (*P *= 0.001, 0.015, respectively). It was established that serum NO and MDA levels of the NAC group were significantly lower than the sham and PAM-atropine groups in the 12th hour (*P *= 0.043, 0.041, respectively). The erythrocyte CE levels of the NAC group in the 24th hour was significantly higher than the PAM-atropine group (*P *= 0.015). The erythrocyte NO and MDA levels of the NAC group in the 24th hour were significant lower than the PAM-atropine group (*P *= 0.037, 0.028, respectively). No significant difference was determined between the NAC group and PAM-atropin group for liver tissue CE and NO levels (*P *= 0.055, 0.109, respectively). The liver tissue MDA levels of the NAC group were significantly lower than the sham and PAM-atropine groups (*P *= 0.004, 0.004, respectively).

## Conclusions

In the treatment of AOP, NAC has a favorable effect on both blood and liver tissue CE activity, NO levels and lipid peroxidation. Adding to antidote treatment of NAC could reduce organ damage, morbidity and mortality. Further clinical studies could be elucidated for this subject.

